# LRRK2 Kinase Inhibitor PF-06447475 Protects *Drosophila melanogaster* against Paraquat-Induced Locomotor Impairment, Life Span Reduction, and Oxidative Stress

**DOI:** 10.1007/s11064-024-04141-9

**Published:** 2024-06-07

**Authors:** Diana A. Quintero-Espinosa, Marlene Jimenez-Del-Rio, Carlos Velez-Pardo

**Affiliations:** Neuroscience Research Group, Medical Research Institute, Faculty of Medicine, University of Antioquia (UdeA), Calle 70 No. 52-21, and Calle 62 # 52-59, Building 1, Room 412, SIU, Medellin, Colombia

**Keywords:** PF06447475, dLrrk2, *Drosophila*, Paraquat, Neurodegeneration, Oxidative stress

## Abstract

Parkinson’s disease (PD) is a complex multifactorial progressive neurodegenerative disease characterized by locomotor alteration due to the specific deterioration of dopaminergic (DAergic) neurons in the substantia nigra pars compacta (SNpc). Mounting evidence shows that human LRRK2 (hLRRK2) kinase activity is involved in oxidative stress (OS)-induced neurodegeneration, suggesting LRRK2 inhibition as a potential therapeutic target. We report that the hLRRK2 inhibitor PF-06447475 (PF-475) prolonged lifespan, increased locomotor activity, maintained DAergic neuronal integrity, and reduced lipid peroxidation (LPO) in female *Drosophila melanogaster* flies chronically exposed to paraquat (PQ), a redox cycling compound, compared to flies treated with vehicle only. Since LRRK2 is an evolutionary conserved kinase, the present findings reinforce the idea that either reduction or inhibition of the LRRK2 kinase might decrease OS and locomotor alterations associated with PD. Our observations highlight the importance of uncovering the function of the hLRRK2 orthologue dLrrk2 in *D. melanogaster* as an excellent model for pharmacological screenings.

**Supplementary Information:**

The online version contains supplementary material available at 10.1007/s11064-024-04141-9.

## Introduction

Parkinson’s disease (PD) is a neurological condition characterized by the progressive loss of 50–70% of dopaminergic (DAergic) neurons from the substantia nigra, leading to movement alterations [1]. PD is now considered a pandemic disease [2]. Therefore, one of the major goals of PD research is to protect this group of neuronal cells from structural and functional deterioration or the dying process. Unfortunately, elucidation of the molecular mechanisms of neuronal loss or damage has been elusive until now [3, 4]. Despite this drawback, genetic and environmental factors have been postulated to play an important role in the development of PD, resulting in oxidative stress (OS), mitochondrial damage, and neuronal loss [5]. Although PD cases are mainly sporadic, about 10% of the cases have a familial origin. The most common genetic form associated with autosomal dominant familiar cases has been linked to the leucine-rich repeat kinase 2 (LRRK2) gene [6, 7]. This gene spans a 144-kb genomic region, with 51 exons encoding a large protein of 2,527 amino acids (aa) with multiple functional domains, including an ankyrin repeat region, a leucine-rich repeat domain, a kinase domain, a RAS domain, a Ras-of-complex-GTPase domain, and a WD40 motif [8]. Although the normal biological function of LRRK2 is not yet fully established, this kinase has been involved in cell survival, OS, and mitochondrial dynamics [9]. Indeed, LRRK2 kinase has been found to be responsible for OS-induced neurotoxicity [10]. In line with these findings, our laboratory group has demonstrated that inhibitor LRRK2, PF-06447475 (referred to here as PF-475; (3-[4-(Morpholin-4-yl)-7 H-pyrrolo[2,3-d]pyrimidin-5-yl]benzonitrile) [11], protected nerve-like differentiated cells against OS-induced apoptosis [12]. However, no data are available to establish the definitive role played by LRRK2 in OS-stimuli in vivo.

Paraquat dication (PQ2+) is chemically named 1-methyl-4-(1-methylpyridin-1-ium-4-yl) pyridin-1-ium, an organic cation that consists of 4,4’-bipyridine bearing two N-methyl substituents located at the 1- and 1’-positions (PubChem Compound CID: 15,939). Despite being a non-selective contact herbicide and highly toxic compound [13], PQ2 + is today among the most used herbicides in the world (e.g., Global Paraquat Market, 2024; https://reports.valuates.com/market-reports/QYRE-Auto-9X753/global-paraquat). Unfortunately, exposure to PQ2 + has been associated with the development of PD (e.g., [14, 15]). As a neurotoxin, PQ2 + works as a redox cycling compound [16]. PQ2 + is reduced enzymatically, mainly by NADH: ubiquinone oxidoreductase (complex I), to form the paraquat monocation (PQ·+), which is then reoxidized in the presence of dioxygen (O2) with subsequent production of superoxide anion radical (O2^.−^). Dismutation of this last molecule generates reactive oxygen species (ROS), leading to several cellular and molecular deleterious events, including oxidative stress (OS), mitochondria dysfunction, alteration in dopamine metabolism, and lipid peroxidation (LPO) [17, 18]. Given those well-characterized features, PQ2 + has been widely used in vivo to create a variety of PD pathological features [19].

*Drosophila melanogaster* (referred to here as *D. melanogaster* or *Drosophila*) is one of the most widely used experimental organisms for neurodegenerative disorders [20]. Specifically, the fly provides an excellent toolkit for the modeling of environmental and genetic aspects of PD [21–23]. Indeed, *D. melanogaster* reproduces many features of the disease, such as loss of DAergic neurons, reduced movement, mitochondrial abnormalities, LPO, and OS [24–26]. Human LRRK2 (hLRRK2) kinase is a highly conserved protein at a structural and functional level across species [27]. *D. melanogaster* has a single orthologue, dLrrk2, and the kinase domain is 31% identical and 52% similar between dLrrk2 and hLRRK2 [28]. Interestingly, studies have shown that genetic ablation of dLrrk2 in *Drosophila* [29, 30] or pharmacological inhibition in rats and worms [31, 32] provide resistance to OS-stimuli. Despite these advances, it is not yet established whether pharmacological inhibition offers the most effective and promising approach to PD management. Since *Drosophila* presents a high percentage identity and similarity of disease-related genetic variants (approximately 75%; [33]) and has deep homology with brain structures involved in PD neuropathology [34, 35], the fly might be an excellent model organism for drug screening [36].

The aim of the present investigation was to assess the effect of the hLRRK2 kinase inhibitor PF-475 in female *D. melanogaster* flies (w[1118]). To this end, flies were chronically exposed to PQ for 15 days in the presence or absence of the inhibitor PF-475 to evaluate lifespan, locomotor activity, integrity of DAergic neurons in terms of TH expression level, and lipid peroxidation (LPO), as indicative of OS. Our findings suggest that dLrrk2 (or hLRRK2) work as regulator of OS and that their action could be blocked by specific inhibitor LRRK2 PF-475. The high similarity between hLRRK2 and dLrrk2 highlights the importance of uncovering the function of the LRRK2 orthologue dLrrk2 in *Drosophila* as an invaluable model for pharmacological screenings.

## Materials and methods

### Fly Stock and Culture

Stock vials of *Drosophila* melanogaster were raised at 25 °C on a 12-hour light/dark cycle in bottles containing Nutri-FlyTM (Flystuff-Genesee Scientific) fly food medium. Propionic acid was added to prevent fungal growth (Merck-Schuchardt OHG D-85,662, Hohenbrunn, Germany). Unless specified otherwise, other reagents were purchased from Sigma (St. Louis, MO, USA). Given that female flies appeared more sensitive to PQ intoxication than males [37], wild type (WT) (Bloomington Drosophila Stock Center (BDSC) #3605, w[1118]) and TH > Lrrk2-RNAi female flies were used for experiments. Directed suppression of dLrrk2 related transgenes was achieved using the Gal4 > UAS system, with line UAS-Lrrk-RNAi (BSC #35,249: y[1]sc[⁄]v[1]; P{y[+ t7.7]v[+ t1.8] = TRiP.GL00136}attP2/TM3,Sb[1]) and TH-Gal4 ((BSC) #8848: w[⁄]; P{w[+ mC] = ple-GAL4.F}3). The sample size was calculated by power analysis according to the formulas: sample size = 2 (Zα /2 + Zβ) 2 × P(1-P)/ (p1 -p2) 2, Zα /2 = Z0.025 = 1.96 (from Z table) at a type I error of 5%; Zβ = Z0.20 = 0.842 (from Z table) at 80% power; p1-p2 = difference in proportion of events in two groups = − 0.4 Previous studies in our group suggested that if PQ (1 mM) is given for 5 days orally to flies, 50% of them will die within this period, hence survival is 50% (0.5 proportion). If PF-475 increases survival to 90% (0.9 proportions), then these findings can be considered significant. Effect size = 0.5–0.9 = − 0.4). Pooled prevalence = 0.5 + 0.9/2 = 0.7. At 5% significance level and 80% power, the sample size will be 20.60 flies (~ 21 flies) per group. This value, if adjusted for 30% attrition, will be 30 flies [38]. To increase statistical power, we thus used *n* = 60 flies per group for protection assays.

### Experimental Procedures

#### Protection Assay

A protection assay was performed on virgin 2- to 3-day-old female flies collected overnight and kept on regular food medium. Subsequently, 60 separated adult female flies were starved in empty vials for 3 h at 25 °C. Then, groups of six flies were placed in ten vials containing a filter paper (Bio Rad Mini Trans-Blot 1,703,932) saturated with 1% glucose (1G, 55.5 mM glucose) in distilled water (dW) for 24 h. After this time, flies were starved in empty vials for 3 h at 25 °C and transferred to vials with a filter paper saturated with 200 µl paraquat (PQ 1 mM, MP Biomedicals cat 02195323) and/or hLRRK2 selective inhibitor PF-475 at 0.05, 0.075, and 0.1 µM (Sigma cat PZ0248) in 1G for 15 days. The PQ feeding schedule was adjusted according to the protection assay. Red food dye (8 µl/1 ml) (Red food color McCormick) was added to ensure homogeneity and food intake. Living flies were counted daily.

### Locomotion Assay

The movement deficit assay was performed on treated flies, according to Ortega-Arellano et al. [39]. Briefly, treated flies were placed in empty plastic vials. After a 10-minute rest period, the flies were tapped to the bottom of the vials, and the number of flies able to climb 5 cm in 6 s was recorded at each interval of time. The assays were repeated three times at 1-minute intervals. For each experiment, a climbing percent (%) was calculated as ½ [(n_tot_ + n_top_ - n_bot_)/n_tot_] x 100. Data were shown as a mean ± standard deviation of the mean (SD). The Chi Square (c^2^) statistic was performed to compare proportions of percentages between independent groups. Differences were considered statistically significant at *p* < 0.05.

### Survival Test

Flies were treated chronically with paraquat (PQ; 1 mM, MP Biomedicals cat 02195323) and the selective inhibitor PF-475 (0.05, 0.075, and 0.1 mM, Sigma cat PZ0248) as described above for 15 days. Live flies were counted in groups of six per vial daily. Sixty flies per treatment were used. Survival curves were plotted using the Kaplan-Meier estimator. The statistical significance was calculated using the log rank test within the portable IBM SPSS Statistics 22 package program. The null hypothesis in all survival assays was that the exposure of PQ and/or PF-475 to female *Drosophila* made no difference in the survival of the flies in the absence of those reagents. Differences were considered statistically significant at *p* < 0.05.

### Western Blotting Analysis

Adult fly heads were homogenized at 4 °C in a lysis buffer (20 mM Tris-HCl (pH 7.5), 150 mM NaCl, 1 mM Na EDTA, 1 mM EGTA, 1% Triton, 2.5 mM sodium pyrophosphate, 1 mM beta-glycerophosphate, 1 mM Na_3_VO_4_, 1 µg/ml leupeptin with protease inhibitor PFMS 1 mM). Samples were placed at − 80 °C for 5 min, centrifuged for 15 min × 13,000 rpm in a cold microcentrifuge, and supernatants were recovered and stored at − 80 °C. The resulting homogenate was subjected to BCA protein assays to ensure equal protein loading, resolved on 8–12% SDS/PAGE Bis-Tris gels, and transferred onto Hybond ECL 0.45 μm Nitrocellulose membranes (GE Healthcare Life Sciences). The membranes were blocked in TBS (pH 7.4, 10 mM Tris-HCl, 150 mM NaCl, 0.1%) containing 5% non-fat milk. Primary antibodies: anti-dLrrk2 (Sigma Aldrich SAB1300356; 1:200 dilution), anti-TH (Millipore ab112; 1:5000 dilution), and anti-actin (Abcam Inc. ab50412; 1:5000 dilution) were incubated overnight in TBS-T (ph 7.4, 10 mM Tris-HCl 150 mM, NaCl 0.1%, Tween 20 0.01%) containing 1.5% non-fat milk. IRDye 800CW Donkey anti-goat or anti-rabbit (LI-COR Biosciences; 1:10,000). Proteins were detected by the Odyssey infrared imaging system (LICOR Biosciences). The WB analysis was assessed (protein/actin ratio (arbitrary units)) three times in independent experiments.

### Lipid Peroxidation (LPO) Assay

Quantification of lipid peroxidation involving TBARS (thiobarbituric acid reactive substance) was performed according [40]. Briefly, 30 heads (approx. 30 mg) from untreated or PQ (1 mM) treated flies with or without PF-475 were homogenized in a 0.6 mL solution containing 50 mM sodium phosphate buffer, pH 6.0, and 10% trichloroacetic acid (TCA). Then, samples were centrifuged at 10,000 rpm for 10 min. The supernatant was divided into two aliquots. The first supernatant (0.3 mL) was mixed with 0.1 mL of 0.1 M EDTA and 0.6 mL of a solution containing 1% thiobarbituric acid in 0.05 M NaOH, and then incubated at 100 ºC for 15 min. The second aliquot (0.3 mL) was mixed with 0.7 mL of H_2_O and incubated under the same conditions as described above. This sample was used as an internal absorbance control to avoid artifacts in the LPO measurement. After cooling on ice, the samples were centrifuged at 10,000 rpm for 1 min. The malondialdehyde (MDA) product was measured at 535 nm. The molar absorptivity of MDA (1.56 × 10^5^ M^− 1^ cm^− 1^) was used to express lipid peroxidation levels as MDA (nMol per mg of fly heads). To compare the differences between two or more groups, a one-way ANOVA followed by the Bonferroni post hoc comparison calculated with SPSS 25 software was performed. Differences were considered statistically significant at *p* < 0.05.

### Molecular Docking

We used X-ray crystal structure of hLRRK2 (protein data bank code: 7LI4) and dLrrk2 (code AF-A0A0B4KHT3, created with Alphafold, https://alphafold.ebi.ac.uk/) for molecular docking experiments. The blind molecular docking was performed with CB-Dock version 2 [41], a cavity detection-guided protein-ligand blind docking web server that uses Autodock Vina (version 1.1.2, Scripps Research Institute, La Jolla, USA) or FitDock: protein-ligand docking by template fitting. The SDF structure files of the tested compounds (ATP, compound CID: 5957, and PF06447475, compound CID: 72,706,840) were downloaded from PubChem (https://pubchem.ncbi.nlm.nih.gov/; available in March 2024). The molecular blind docking was performed by uploading the 3D structure PDB file of hLRRK2 and dLrrk2 into the server with the SDF file of each compound. For analysis, we selected the docking poses with the strongest Vina score or fitDock score in the catalytical pocket. The generated PDB files of the molecular docking of each compound were visualized with the CB-Dock2 interphase or through BIOVIA Discovery Studio Visualizer (https://discover.3ds.com/discovery-studio-visualizer-download; available in March 2024) and compared against the experimentally validated X-ray structures of the interaction of ATP with hLRRK2 [42].

## Results

### PF06447475 (PF-475) Binds with High Affinity to the ATP-Binding Pocket of the dLrrk2 Kinase in *D. melanogaster*

**Fig. 1:**
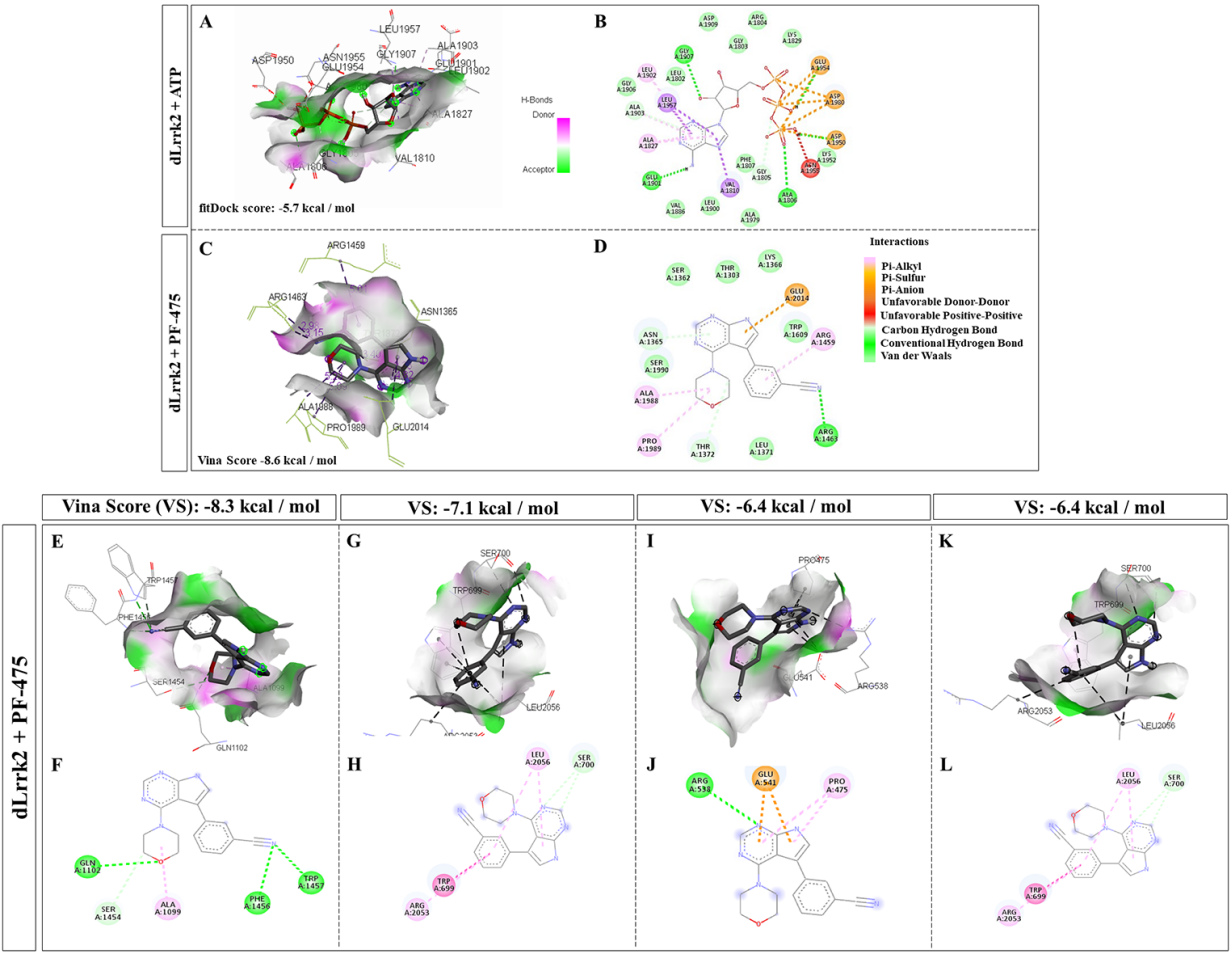
LRRK2 inhibitor ATP and PF-06447475 bind to the active ATP pocket of dLrrk2. (**A)** Representative CB-Dock2 three-dimensional (3D) images of ATP (Compound CID: 5957) with the hydrogen bond donor-acceptor residues and hydrophobic surface pocket in dLrrk2 kinase (Vina Score − 5.7 kcal/mol); and (**B)** 2D schematic diagram of docking model of ATP bound to the pocket in dLrrk2 kinase (AF-A0A04KHT3-F1); (**C)** Representative CB-Dock2 three-dimensional (3D) images of PF06447475 (Compound CID: 72,706,840) with the hydrogen bond donor-acceptor residues and hydrophobic surface pocket in dLrrk2 kinase (Vina Score (VS) -8.6 kcal/mol); (**D)** 2D schematic diagram of docking model of PF06447475 bound to the pocket in dLrrk2 kinase. Representative CB-Dock2 three-dimensional (3D) images of PF06447475 bound to the dLrrk2 kinase pocket (**E)** with a VS -8.3 kcal/mol, (**G**) VS -7.1 kcal/mol, and (**I, K)** VS -6.4 kcal/mol. Representative 2D schematic diagram of docking model of PF06447475 bound to the dLrrk2 kinase pocket with a with a (**F**) VS -8.3 kcal/mol, (**H**) VS -7.1 kcal/mol, and (**J, L)** VS -6.4 kcal/mol. Residues involved in hydrogen bonding, charge, polar, or van der Waals interactions are represented by respective color indicated in inset of the figure

We first investigated whether PF-475 might bind to the ATP pocket of the dLrrk2 kinase in *D. melanogaster*, as it has previously been reported in the hLRRK2 kinase [11]. As a validation procedure, we performed an in silico molecular docking analysis using CB-Dock2, an accurate protein-ligand blind docking tool [41], to analyze the ATP-binding and PF-475-binding pockets in hLRRK2. As expected, docking analysis reveals that ATP and PF-475 are bound to hLRRK2 with high affinity, albeit with different strengths. ATP bound to the dLRRK2 pocket with an affinity of -5.8 kCal/mol (fitDock score), involving meanly 21 aa (Table 1, Suppl Fig. S1A-B), whereas PF-475 inhibitor bound to the hLRRK2 pocket with an affinity of -6.9 kCal/mol (fitDock score), involving nearly the same aa in the catalytical pocket, 17/21 (81%) (Table 1, Suppl Fig. S1C-D). Indeed, the PF-475 inhibitor shows 1.19 times higher affinity for hLRRK2 than ATP. Similarly, molecular docking analysis shows that PF-475 bound to the hLrrk2 pocket with a higher affinity score (-8.6 kcal/mol, Vina score) than ATP (-5.7 kcal/mol, fitDock score) (Table 1; Fig. 1A-D). These theoretical calculations indicated the presence of a PF-475 high affinity binding pocket in the dLrrk2 kinase (Table 1; Fig. 1E-L).

**Table 1:** In silico molecular docking analysis of human LRRK2 (7LI4) or Drosophila Lrrk2 (AF-A0A0B4KHT3) with ATP or PF-475

Submitted Protein PDB*	Submitted Ligand**	Vina Score*** fitDock score	Cavity volume (Å^3^)	Center (x, y, z)	Docking size (x, y, z)	Contact residue [ref.]
**7LI4**	ATP (Compound CID: 5957)	**-5.8**	6878	174,214,200	35,35,35	Chain A: **ASP1887 GLY1888 SER1889 GLY1891** SER1892 VAL1893 ALA1904 LYS1906 ILE1933 **MET1947 GLU1948 LEU1949 ALA1950 GLY1953** SER1954 **ARG1957** LYS1996 HIS1998 ASN1999 LEU2001 TYR2018 [42]
	PF06447475 (Compound CID: 72,706,840)	**-6.9**	6878	174,214,200	35,35,35	Chain A: **ASP1887 GLY1888 SER1892 VAL1893 ALA1904** LYS1906 ILE1933 **MET1947 GLU1948 LEU1949 ALA1950 GLY1953** SER1954 **HIS1998 LEU2001 ALA2016 TYR2018**
**AF-A0A0B4KHT3**	ATP (Compound CID: 5957)	**-5.7**	4773	20,-9, 2	21, 35, 35	Chain A: LEU1802 GLY1803 ARG1804 GLY1805 **ALA1806** PHE1807 GLY1808 **VAL1810 ALA1827** LYS1829 VAL1886 LEU1900 **GLU1901 LEU1902 ALA1903** GLY1906 **GLY1907** ASP1909 **ASP1950 LYS1952** **GLU1954 ASN1955 LEU1957** TRP1959 ALA1979 **ASP1980** THR1998
	ATP (Compound CID: 5957)	**-4.9**	4773	20,-9, 2	21, 35, 35	Chain A: LEU1802 GLY1803 ARG1804 GLY1805 **ALA1806 VAL1810 ALA1827** LYS1829 VAL1886 LEU1900 **GLU1901 LEU1902 ALA1903** GLY1906 **GLY1907** ASP1909 **ASP1950 LYS1952 GLU1954 ASN1955** **LEU1957** TRP1959 ALA1979 **ASP1980**
	ATP (Compound CID: 5957)	**-4.6**	4773	20,-9, 2	21, 35, 35	Chain A: LEU1802 GLY1803 ARG1804 GLY1805 **ALA1806** GLY1808 **VAL1810 ALA1827** LYS1829 GLU1873 LEU1900 GLU1901 LEU1902 ALA1903 GLY1906 GLY1907 **ASP1909 GLU1954 ASN1955** VAL1956 LEU1957 TRP1959 ALA1979 **ASP1980** TYR1981
	ATP (Compound CID: 5957)	**-3.8**	4773	20,-9, 2	21, 35, 35	Chain A: LEU1802 GLY1803 ARG1804 GLY1805 **ALA1806 VAL1810 ALA1827** LYS1829 VAL1886 LEU1900 GLU1901 LEU1902 ALA1903 GLY1906 GLY1907 **ASP1909 ASP1950 LYS1952 GLU1954 ASN1955** **LEU1957** TRP1959 ALA1979 **ASP1980** THR1998
	ATP (Compound CID: 5957)	**80.3**				Chain A: LEU1802 ARG1804 GLY1805 **ALA1806** PHE1807 GLY1808 **VAL1810 ALA1827** LYS1829 LEU1831 THR1869 GLU1873 VAL1886 LEU1900 **GLU1901 LEU1902 ALA1903** ASP1950 **GLU1954 ASN1955** **LEU1957** TRP1959 ALA1979 **ASP1980** TYR1981 GLY1982 ILE1983
	PF06447475 (Compound CID: 72,706,840)	**-8.6**	4773	20,-9,2	20,35,35	Chain A: ASN1365 LYS1366 LEU1371 THR1372 TRP1373 ASP1374 ARG1459 THR1462 **ARG1463** ALA1466 ASP1608 TRP1609 ALA1988 PRO1989 SER1990 GLU2014
	PF06447475 (Compound CID: 72,706,840)	**-8.3**	4087	35, 6, -9	20,35,35	Chain A: LYS1095 TYR1098 ALA1099 **GLN1102** TYR1103 GLN1129 PRO1381 SER1382 PRO1453 SER1454 GLY1455 **PHE1456 TRP1457** SER1458 GLN1503 THR1504
	PF06447475 (Compound CID: 72,706,840)	**-7.1**	1179	-23, 34, 8	20,20,20	Chain A: ASP709 MET710 LYS711 TRP712 PRO749 VAL753 ASN754 PRO775 ALA776 THR777 ARG845 HIS846
	PF06447475 (Compound CID: 72,706,840)	**-6.4**	2197	-56, -2, -12	20, 29,20	Chain A: PRO475 GLU479 **ARG538** GLU541 VAL542 LEU544 THR545 GLU563 HIS566 LEU567 VAL568
	PF06447475 (Compound CID: 72,706,840)	**-6.4**	3079	-15, 2, 18	20, 35, 32	Chain A: LEU698 TRP699 SER700 THR702 LEU703 SER2052 ARG2053 PRO2054 ALA2055 LEU2056 THR2061 MET2071 VAL2072 TRP2075

* According to RCSB Protein Data Base (https://www.rcsb.org/)

**According to PubChem database (https://pubchem.ncbi.nlm.nih.gov/)

*** According to CB-dock2: An accurate protein-ligand bind cocking tool https://cadd.labshare.cn/cb-dock2/php/index.php

The fitDock score is based on *Template-based docking* [63]

Bold letters represent important amino acid residue in the (catalytic) protein pocket that interact with ATP or hLRRK2 inhibitor PF-475

### PF-06447475 Increases Lifespan and Reduces Motor Impairment in Flies Exposed to Paraquat

**Fig. 2:**
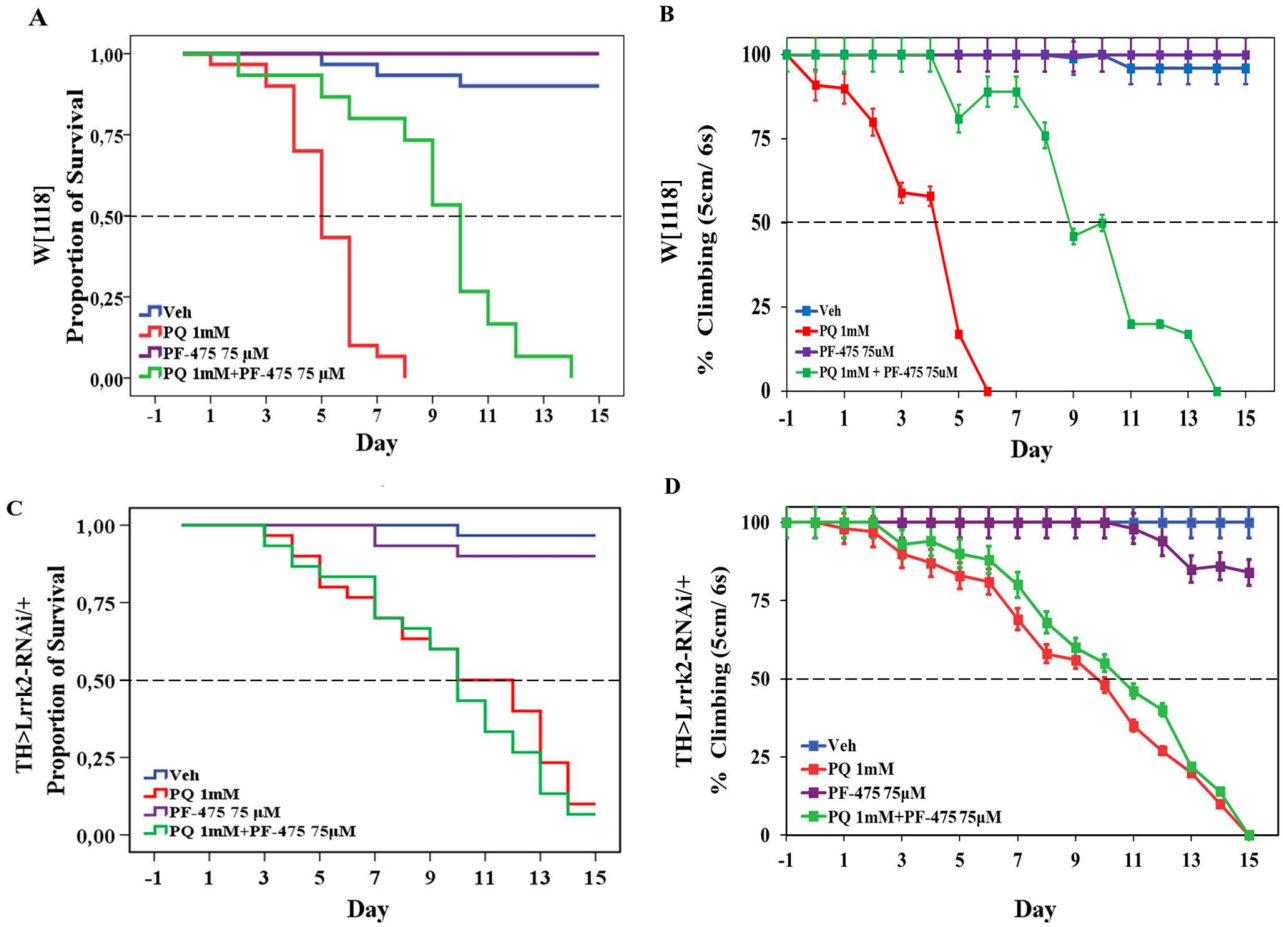
PF-06447475 prolongs life span and locomotor activity in Drosophila flies chronically exposed to PQ. Female flies (*n* = 60 per treatment) were left untreated or treated with PQ, PF-06447475, or PQ and PF-06447475 as is described in *Materials and Methods*. The graphs show (**A**) the survival and (**B**) climbing analysis of flies. The PF-06447475 increases the survival index and percentage of climbing of *Drosophila* in an average of 5 days compared to the flies treated with PQ only. The graphs show (**C**) the survival and (**D**) climbing analysis of TH > Lrrk2-RNAi/+ flies untreated or treated with PQ in absence or presence of PF-06447475. Statistical comparisons between untreated and treated flies showed (**A**, **C**) *p* < 0.05 by log-rank test and (**B**, **D**) *p* < 0.05 by χ^2^ test

Previously, it has been demonstrated that PQ (1 mM) induced a significant reduction in survival and locomotor activity in TH/+ flies and such noxious effects were importantly diminished in TH > Lrrk2-RNAi/+ flies [30]. Effectively, w[1118] flies fed with PQ (1 mM) had a significantly decreased lifespan (Fig. 2A) and locomotor activity (Fig. 2B) compared to untreated flies. Noticeably, 50% of w[1118] flies exposed to PQ perished at day 5 and reduced locomotor activity at the same day, but survival and locomotor activity in flies treated with vehicle only were extended beyond day 15 (Table 2, track 1 vs. 2, Fig. 2A and B). We confirmed that TH > Lrrk2-RNAi/+ flies exposed to PQ showed a prolonged survival (Fig. 2C) and increased percentage of climbing activity (Fig. 2D). Of note, whereas 50% of TH > Lrrk2-RNAi/+ flies treated with vehicle only extended the survival and locomotor activity beyond day 15 (Table 2, track 11, Fig. 2C and D), the knockdown flies exposed to PQ perished at day 10 and reduced locomotor activity at the same day (Table 2, track 12, Fig. 2C and D).

**Table 2:** *Drosophila* flies treated with PF06447475 are resistant to PQ-induced life span decrease and locomotor impairment

Line	Noxious or Treatment	#	Concentration (mM)	Effect on							
				Survival (Day)^a^			K-M, *p*	Climbing (Day)^b^			χ^2^, *p*
				**25%**	**50%**	**75%**		**25%**	**50%**	**75%**	
w[1118]	PQ	**1**	**0**	**> 15**	**> 15**	**> 15**	**1 vs. 2, p < 0.005**	**> 15**	**> 15**	**> 15**	**1 vs. 2, p < 0.005**
		**2**	**1**	**4**	**5**	**6**	**2 vs. 1, p < 0.005**	**2**	**4**	**5**	**2 vs. 1, p < 0.005**
PF-475		3	0.025	> 15	> 15	> 15	3 vs. 1, n.s.	> 15	> 15	> 15	3 vs. 1, n.s.
		4	0.050	> 15	> 15	> 15	4 vs. 1, n.s.	> 15	> 15	> 15	4 vs. 1, n.s.
		**5**	**0.075**	**> 15**	**> 15**	**> 15**	**5 vs. 1, n.s.**	**> 15**	**> 15**	**> 15**	**5 vs. 1 n.s.**
		6	0.100	6	7	8	6 vs. 1, *p* < 0.005	6	7	8	6 vs. 1, *p* < 0.005
PQ + PF-475		7	1 + 0.025	6	7	9	7 vs. 2, n.s.	5	6	8	7 vs. 2, n.s.
		8	1 + 0.050	7	8	9	8 vs. 2, *p* < 0.05	6	8	9	8 vs. 2, *p* < 0.05
		**9**	**1 + 0.075**	**8**	**10**	**11**	**9 vs. 2, p < 0.005**	**8**	**9**	**11**	**9 vs. 2, p < 0.005**
		10	1 + 0.100	2	4	6	10 vs. 2, n.s.	2	4	6	10 vs. 2, n.s.
*TH > Lrrk-RNAi/+*	PQ	11	0	**> 15**	**> 15**	**> 15**	**11 vs. 1, n.s.**	**> 15**	**> 15**	**> 15**	**11 vs. 1, n.s.**
		12	1	**7**	**10**	**13**	**12 vs. 2, p < 0.005**	**8**	**10**	**12**	**12 vs. 2, p < 0.005**
PF-475		13	0.075	**> 15**	**> 15**	**> 15**	**13 vs. 5, n.s.**	**> 15**	**> 15**	**> 15**	**13 vs. 5, n.s.**
PQ + PF-475		14	1 + 0.075	**7**	**10**	**14**	**14 vs. 2, n.s.**	**7**	**10**	**14**	**14 vs. 12, n.s.**

Letters and numbers in bold represent data shown in Fig. 2

^a^ Represents number of days in which 25%, 50%, and 75% of total flies have been killed

^b^ Represents number of days in which 25%, 50%, and 75% of climbing ability is impaired

* *P* ≤ 0.005

*Abbreviations*. PQ, Paraquat; PF-475: LRRK2 kinase inhibitor PF-06447475; K-M, Kaplan-Meier test; n.s.: no significance; *TH > Lrrk-RNAi/+: Tyrosine hydroxylase, GAL4 > Lrrk-RNAi/+;* χ^2^, Chi-square test

We next evaluated the effect of PF-475 alone or in combination with PQ. We initially fed w[1118] flies with increasing concentrations of the inhibitor PF-475 (25, 50, 75, and 100 µM). As described in Table 2, PF-475 at 25–75 µM was innocuous to flies (Table 2, tracks 3–5), whereas 100 µM PF-475 significantly reduced survival and climbing activity in flies (Table 2, track 6) compared to those treated with vehicle only (Table 2, track 1). Inhibitors increased survival and locomotor performance in a concentration-dependent fashion up to 100 µM. Therefore, we selected PF-475 (75 µM) for further experiments. Indeed, flies treated with PF-475 (75 µM) and PQ (1 mM) showed a dramatic increase in life span (Fig. 2A) and climbing activity (Fig. 2B). Remarkably, PF-475 (75 µM) increased almost 2-fold survival and locomotor activity, i.e., 50% treated flies with PQ only at day 5 versus 50% treated flies with PF-475 + PQ at day 9 (~ 45% increase). Interestingly, TH > Lrrk2-RNAi/+ flies treated with PF-475 (75 µM) only (Table 2, track 13) showed a comparable survival and climbing activity to untreated flies (Table 2, track 11), whereas PF-475 co-administered with PQ (Table 2, track 14) showed comparable survival and climbing activity to flies treated with PQ only (Table 2, track 12).

### PF-06447475 Does Not Modify the Expression Level of Tyrosine Hydroxylase (TH) or dLrrk2 Protein in Wild-Type Flies Exposed to Paraquat

**Fig. 3:**
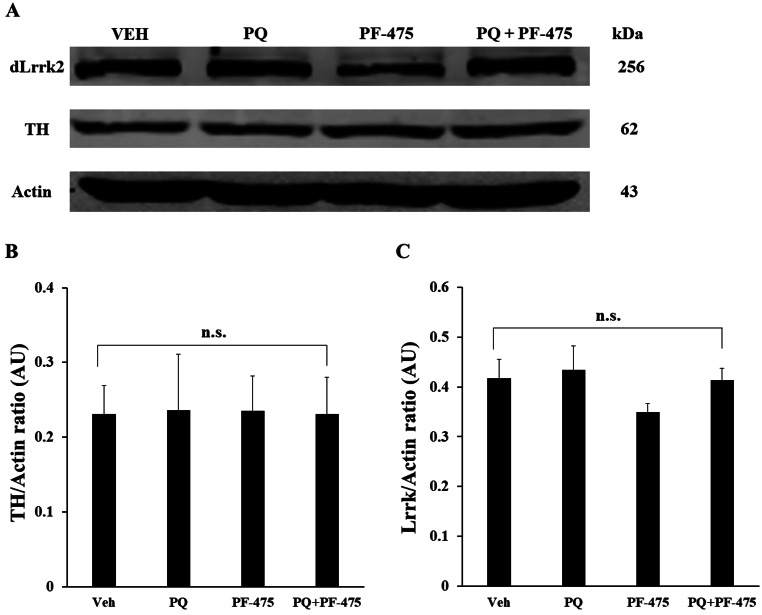
Expression analysis of dLrrk2 and TH protein by Western blot in w[1118] *Drosophila* flies. Protein extracts from females flies (*n* = 60 per treatment) untreated or treated with PF-06447475 in presence or absence of PQ were blotted with primary antibody against dLrrk2, anti-TH, and anti-actin antibody. The intensities of the bands in western blotting (**A**) were measured (**B** and **C**) by an infrared imaging system (Odyssey, LI-COR), and the intensity was normalized to that of actin. **p < 0.05, significant differences between transgenic and control line in each treatment. Error bars indicate ± SD

We wanted to evaluate whether inhibitor PF-475 affected the expression of enzyme TH or dLrrk2 kinase in the absence or presence of PQ. Western blot analysis (Fig. 3A) revealed no statistically significant differences in the expression levels of TH or dLrrk2 (Fig. 3B and C) in flies treated with vehicle only (Fig. 3A, track 1), treated with PQ only (Fig. 3A, track 2), with inhibitor only (Fig. 3A, track 3), or flies treated with both inhibitor and PQ (Fig. 3A, track 4).

### LRRK2 Inhibitor PF-06447475 Reduced the Lipid Peroxidation (LPO) Index in Flies Exposed to Paraquat

**Fig. 4:**
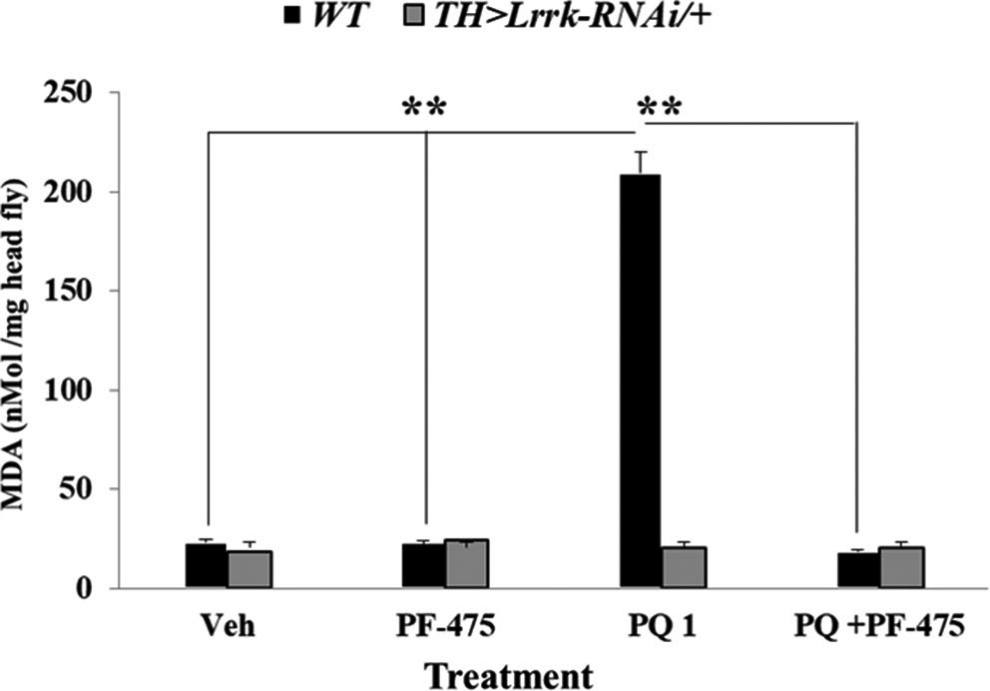
PF06447475 reduces LPO index in w[1118] flies exposed to PQ. Female flies were treated as is described in *Materials and Methods*. The graph shows MDA (Malondialdehyde) concentration as a measure of LPO index. The molar absorptivity of MDA (1.56 × 10 ^5^ M^− 1^ cm^− 1^) was used to express lipid peroxidation levels as nMol of MDA per mg of head flies. The letter n represents the number of fly heads examined per treatment (*n* = 30). **p < 0.05, significant differences between transgenic and control line in each treatment. Error bars indicate ± SD

The above observations prompted us to assess whether PF-475 could reduce PQ-induced OS in flies. As shown in Fig. 4 and Table 3, WT *Drosophila* (w[1118]) flies exposed to PQ produced a statistically increase (9-fold) in MDA compared to flies treated with vehicle only (Table 3, track 1 vs. 2). Remarkably, PF-475 significantly reduced the levels of MDA in flies treated with PQ to a comparable level to flies treated with inhibitor alone or with vehicle only (Table 3, track 1, 3 and 4). Of note, untreated knockdown flies (Table 3, track 5) or treated with PQ (Table 3, track 6), PF-475 only (Table 3, track 7), or PQ and PF-475 (Table 3, track 8) showed similar MDA values (Fig. 4).

**Table 3:** LRRK2 kinase inhibitor PF0647475 reduces LPO index of w[1118] flies chronically exposed to paraquat

Line	Noxious or Treatment	#	Concentration (mM)	Effect on		
				Absorbance	LPO nMol MDA/mg head of fly	ANOVA, *p*
	PQ	1	0	0,018	23	1 vs. 2, *p* < 0.05
w[1118]		2	1	0,111	209	2 vs. 1, *p* < 0.05
	PF-475	3	0.75	0,011	23	3 vs. 1, n.s.
	PQ + PF-475	4	1 + 0.75	0,009	19	4 vs. 2, *p* < 0.05
*TH > Lrrk2 (RNAi)+/-*	PQ	5	0	0,0100	18	5 vs. 1, n.s.
		6	1	0,0112	21	6 vs. 2, *p* < 0.05
	PF-475	7	0.75	0,0132	24	7 vs. 3, n.s.
	PQ + PF-475	8	1 + 0.75	0,0110	20	8 vs. 4, n.s.

*Abbreviations*. Paraquat, PQ; LRRK2 kinase inhibitor PF0647475, PF-475; MDA, Malondialdehyde; LPO, Lipoperoxidation; n.s., no significance; *TH > Lrrk-RNAi/+: Tyrosine hydroxylase, GAL4 > Lrrk-RNAi/+;* ANOVA test

## Discussion

Parkinson’s disease is a complex disease mainly characterized by movement disorder associated with a selective degeneration of DAergic neurons [1] believed to deteriorate by the interaction of genes (e.g., LRRK2) and environmental factors (e.g., exposure to neurotoxin PQ) [43]. However, the gene-environmental interplay in PD is not yet fully understood. Remarkably, *D. melanogaster* has become a simple and powerful system to model PD and understand the pathophysiology of the disorder [44]. Here, we confirmed that *Drosophila* flies chronically exposed to PQ induce a shortage of life span, promote significant high levels of MDA as an indication of LPO and OS, and provoke locomotor impairment as an indication of functional degeneration of DAergic neurons [26, 30, 45]. Taken together, these observations suggest that PQ might be a potential neurotoxin to model *Drosophila* PD-like symptoms [19]. In fact, the PQ-*Drosophila* model has been used to interrogate potential neuroprotective compounds from natural resources (e.g., [46–49]). We also confirmed that TH > Lrrk-RNAi/+ flies treated with PQ showed an extended lifespan and locomotor activity by 100% and 125%, respectively compared to w[1118] flies treated with PQ. We conclude that reduced expression of Lrrk2 in the transgenic TH > Lrrk-RNAi/+ flies conferred resistance PQ stimuli [30].

Given that LRRK2 kinase increases the generation of ROS and causes enhanced neurotoxicity under stress stimuli in dopaminergic SN4741 cells [10] and in neuron-like cells [12], it is therefore reasonable to think that its specific inhibition might alleviate DAergic neurons from OS-induced neurodegeneration in vivo. In the present study, we show for the first time that the inhibitor LRRK2 kinase PF-06447475 (PF-475) attenuates chronic PQ-induced neurotoxicity in *D. melanogaster* by increasing life span, improving climbing ability, and decreasing OS. Our observations suggest that the dLrrk2 kinase modulates neurodegeneration in PD. Although the underlying mechanisms by which dLrrk2 might contribute to PQ-induced neurodegeneration are not yet fully described in the fly, we speculate that it most probably involves ROS-signaling mechanisms [12, 48, 50] and/or alterations of neurocytoskeletal proteins (e.g., [51–53]). Therefore, PF-475 might block dLrrk2, thereby maintaining locomotor activity, reducing OS (LPO index), and prolonging life span, indicative of normal DAergic neuronal functionality. Interestingly, neither PQ alone nor in combination with PF-475 affected the expression levels of TH or dLrrk2. Taken together, these results imply that PF-475 is capable of protecting *Drosophila* against PQ-induced neurodegeneration [30, 45]. These observations suggest that switching off hLRRK2 and dLrrk2 by specific PF-06447475 might be an achievable therapeutic approach in sporadic as well as familial PD.

PF-475 is a second-generation LRRK2 inhibitor with high potency, selectivity, and good blood-brain barrier (BBB) permeability properties in mice and rats [11]. Recent cryo-electron microscopy (cryoEM) analysis of the hLRRK2 protein (resolution 3.10 Armstrong) has provided the means to delve into the structure-function interaction between hLRRK2, its natural ligand ATP, and the inhibitor PF-475 [8]. By using in silico molecular docking analysis, we found that PF-475 binds to hLRRK2 (PDB: 7LI4) with a higher bonding affinity than ATP (1.189-fold increased), involving 16/21 (76%) identical aa residues in the binding pocket of the kinase (Table 1). Interestingly, by using mammalian STE20-like protein kinase 3 (MST3, residues 1-303; NP_001027467; PDB ID: 4U8Z; ATP-binding site residue shows 73% similarity to human LRRK2) as a surrogate crystallographic system for hLRRK2, Henderson and co-workers [11] determined ASP1887, VAL1893, LYS1906, GLU1948, and ALA1950 as important aa residues in the interaction with PF-475. Similarly, we found identical aa interacting in the pocket of hLRRK2 and the inhibitor (Table 1, Suppl. Fig. s2).﻿ Interestingly, a BLAST analysis revealed a 30% identity and 48% similarity between the full length in aa residues of protein dLrrk2 (Uniprot Q9VDJ9|LRRK2_DROME, length 2,445) and hLRRK2 (Uniprot Q5S007|LRRK2_HUMAN, length 2,527). Moreover, a similar BLAST analysis showed a 34% identity and 51% similarity between the kinase domain aa residues of dLrrk2 (Uniprot Q9VDJ9, location 1,728-2,034) and hLRRK2 (Uniprot Q5S007, location 1,879-2,138), including an identical matched sequence at the ATP binding site (dLrrk2: ^1911^KIADYGI^1917^; hLRRK2: ^2015^KIADYGI^2021^) according to software tool FIMO (Find Individual Motif Occurrences [54]. Since PF-475 binds to the ATP pocket in the kinase domain through a DXG motif [55, 56], it is reasonable to think that PF-475 operates as an effective blocker of dLrrk2 through a similar molecular interaction with the human DXG motif. Not surprisingly, PF-475 bound to the binding pocket of dLrrk2 with a higher affinity than ATP (-5.7 vs. -8.6 fitDock score). Taken together, our findings suggest that both hLRRK2 and dLrrk2 are biologically and functionally equivalent. Therefore, the use of Drosophila as an invaluable surrogate in vivo PD model to investigate other potential LRRK2 inhibitors either from natural products (e.g., [57]) or chemical libraries (e.g., [58]) in a simple, fast, reliable, and inexpensive manner. Provided that PF-475 (and other kinase inhibitors) interact with mammalian (hLRRK2) or invertebrate (dLrrk2) kinase in a similar molecular mechanism, we anticipate that pharmacological inhibitors might effectively inhibit LRRK2 in PD patients [59, 60]. Therefore, we anticipate that the use of flies with LRRK2 mutations associated with familial PD (e.g., G2019S, BDSC#602459) might increase the success of finding an effective inhibitor of hLRRK2 for the treatment of PD.

Previous studies have shown that reduced expression of dLrrk2 in the DAergic neurons of transgenic flies conferred PQ resistance and the absence of neurodegeneration [28–30]. Specifically, it has been shown that transgenic flies dramatically increased locomotor activity, reduced the lipid peroxidation (LPO) index alone or in the presence of PQ, presented an extended life span, and showed DAergic neuron integrity and/or functionality. Our present investigation shows similar observations when w[1118] flies were treated with PF-475 and PQ. As expected, PF-475 was inoperant in knockdown flies. Indeed, TH > Lrrk2-RNAi/+ flies exposed to PF-475 and PQ behave similarly as knockdown flies treated with PQ alone. Taken together, these observations suggest that both knockdown and pharmacologic models are biologically comparable and that reduced or suppressed LRRK2 expression by pharmacologic or gene LRRK2 blockage might delay or prevent motor symptoms in persons at risk of suffering Parkinsonism by impeding structural or functional impairment (= neurodegeneration) in the DAergic neurons. However, the pharmacology approach might be ideal over gene therapy for clinical trials.

## Conclusion

LRRK2 is a serine-threonine kinase involved in multiple cellular processes and signaling pathways in PD [61]. Therefore, inhibition of LRRK2 kinase function is a promising therapeutic strategy for PD treatment [62]. Here, we demonstrate that the small-molecule kinase inhibitor PF-06447475 can protect *Drosophila* against OS-induced neurodegeneration. Since *Drosophila* might be biologically homologous to mammalian model organisms (e.g., mice, rats), the fly is an important resource to test and discover new therapeutic compounds. The present *Drosophila*-PQ model might serve as a reference assay, which, together with in silico data results from chemical libraries and/or natural compounds, offers a unique opportunity to speed up the development of novel LRRK2 inhibitors.

### Electronic Supplementary Material

Below is the link to the electronic supplementary material.

Supplementary Material 1

Supplementary Material 2

Supplementary Material 3

Supplementary Material 4

Supplementary Material 5

## Data Availability

No datasets were generated or analysed during the current study.
