# Supplementary figures and images for: LRRK2 Kinase Inhibitor PF-06447475 Protects Drosophila melanogaster against Paraquat-Induced Locomotor Impairment, Life Span Reduction, and Oxidative Stress
Source: Neurochem Res. 2024 Jun 7;49(9):2440–52. doi: 10.1007/s11064-024-04141-9 (PMC11310290; doi:10.1007/s11064-024-04141-9)

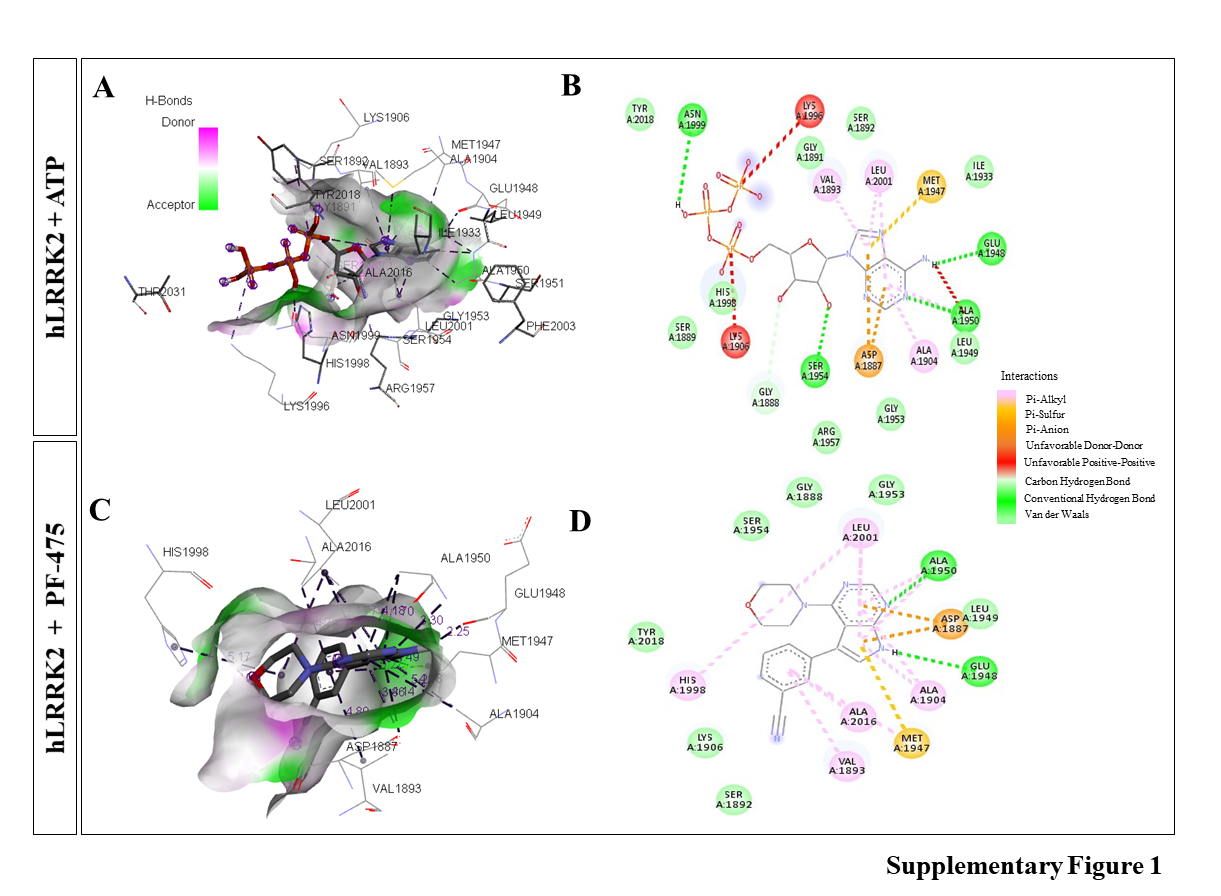

Supplement: Supplementary file 1 — Supplementary Material 1 [file 11064_2024_4141_MOESM1_ESM.tif]

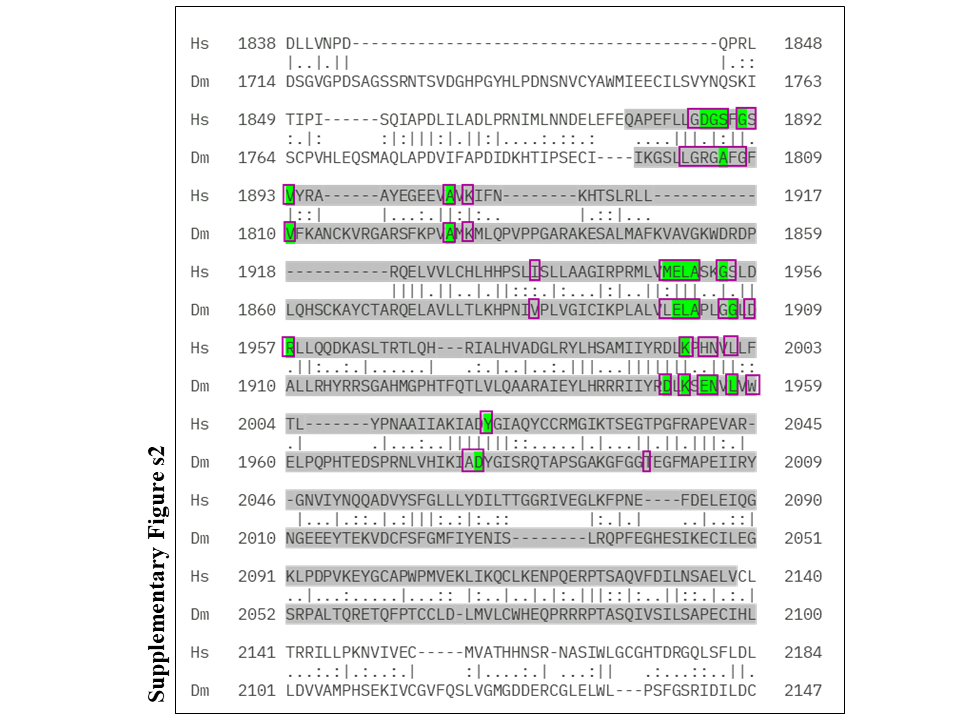

Supplement: Supplementary file 2 — Supplementary Material 2 [file 11064_2024_4141_MOESM2_ESM.tif]

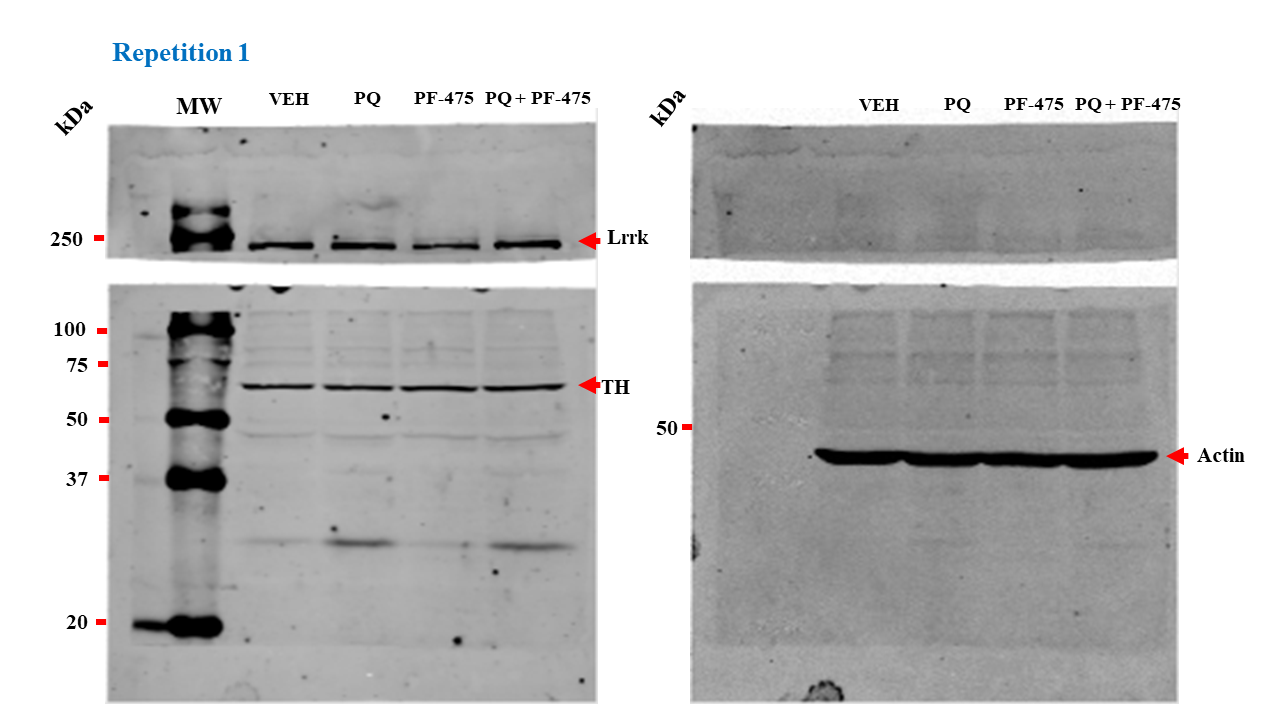

Supplement: Supplementary file 3 — Supplementary Material 3 [file 11064_2024_4141_MOESM3_ESM.tif]

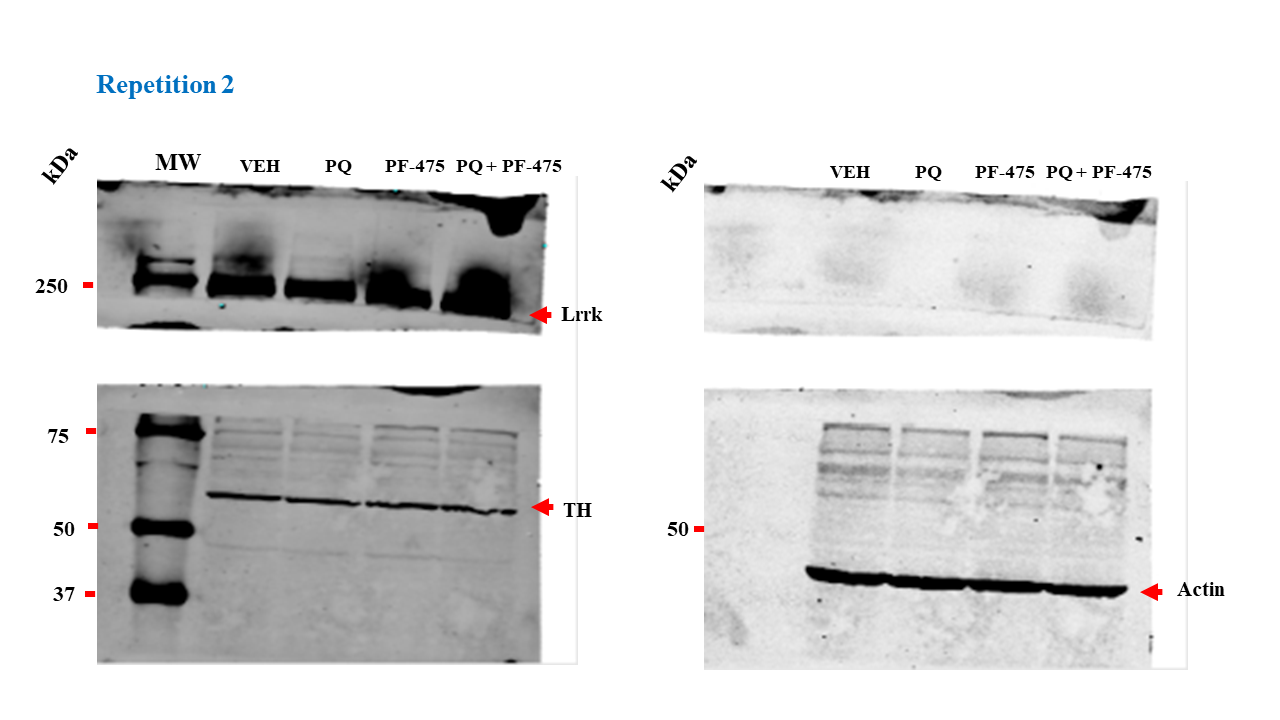

Supplement: Supplementary file 4 — Supplementary Material 4 [file 11064_2024_4141_MOESM4_ESM.tif]

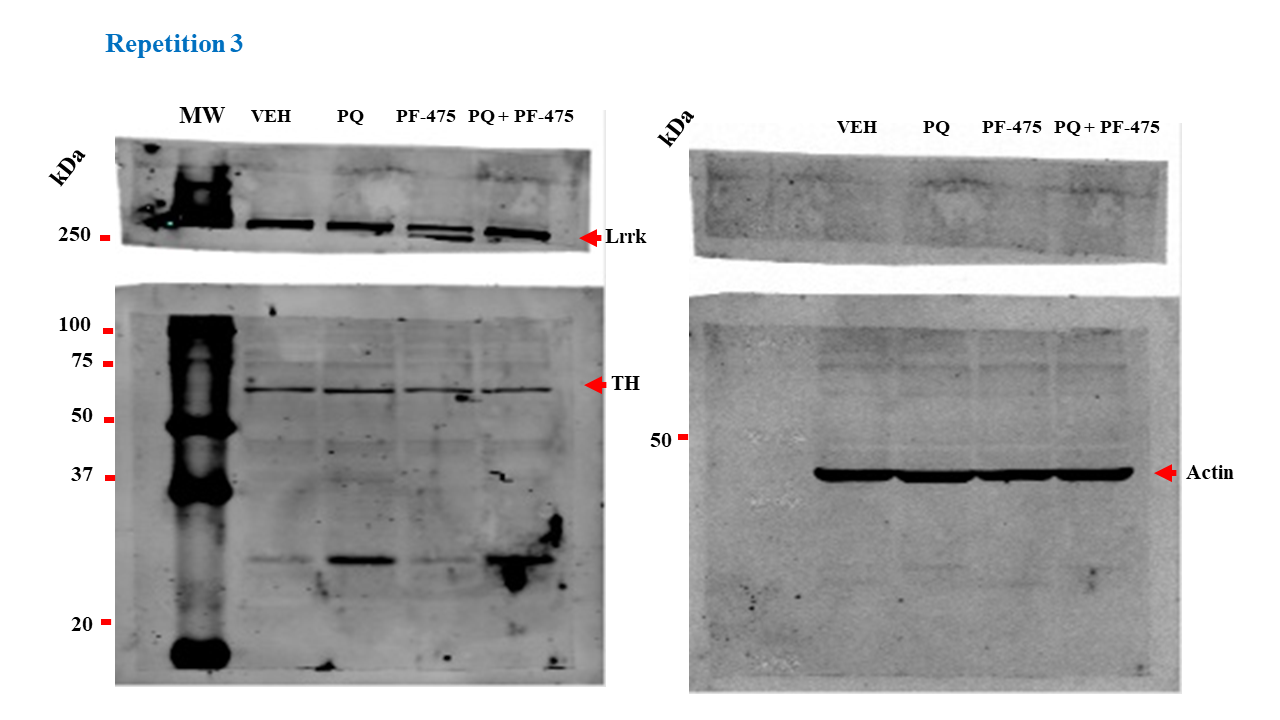

Supplement: Supplementary file 5 — Supplementary Material 5 [file 11064_2024_4141_MOESM5_ESM.tif]
